# Simulation of hemispherical transducers for transcranial HIFU treatments using the hybrid angular spectrum approach

**DOI:** 10.1186/2050-5736-3-S1-P9

**Published:** 2015-06-30

**Authors:** Scott Almquist, Dennis Parker, Douglas Christensen

**Affiliations:** 1 University of Utah, Salt Lake City, Utah, United States

## Background/introduction

Transcranial focused ultrasound is emerging as a promising treatment modality for a variety of disorders including essential tremor and glioblastoma. However, traditional full-wave methods of simulating ultrasound pressure fields for these treatments, such as FDTD, are computationally intensive. The situation is exacerbated by the large area insonified by the transcranial transducers, which are typically hemispherical to spread the intensity over a large area of the skull and prevent burns. The Hybrid Angular Spectrum (HAS) approach [1] has been used to rapidly model ultrasonic beam propagation.

Here we present a method for adapting the HAS technique to hemispherical transducers.

## Methods

The HAS approach assumes that the pressure pattern incident on the front face of the 3D rectangular model is defined on a plane nominally perpendicular to the propagation axis of the transducer. This is problematic for a hemispherical transducer that wraps around the model. To circumvent this limitation, we divide the transducer into seven sections of spatially clustered elements. For each section, the elements, along with the acoustic model, are rotated into a coordinate system with the direction of propagation away from the center of the section. The HAS method is applied using the specific elements of the section, then the resulting pressure pattern is rotated and interpolated back to the original coordinate frame. The fully insonified field is the superposition of the resulting pressure patterns from each section. The resulting field can be used in temperature simulations or to verify the usefulness of phase corrections. Simulations of a 1024-element 650-kHz InSightec ExAblate transducer were carried out using this method on a selected set of retrospective clinical data. The acoustic speed of sound, density and attenuation values for the skull were derived from a CT scan with 0.43 x 0.43 x 1.0-mm resolution using a previously published method of conversion.[2] The simulated skull/brain model contained 512 x 512 x 195 voxels.

## Results and conclusions

**Figure 1:**
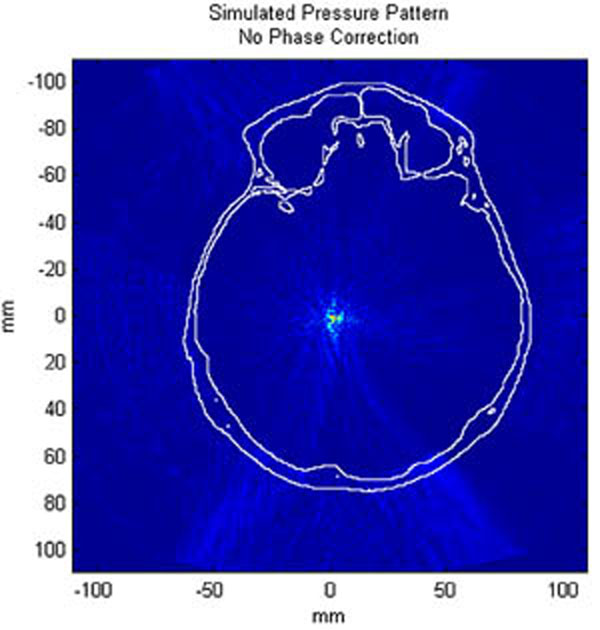
Transverse slice of simulated pressure pattern through the skull with no phase correction employed. Skull is outlined in white

**Figure 2:**
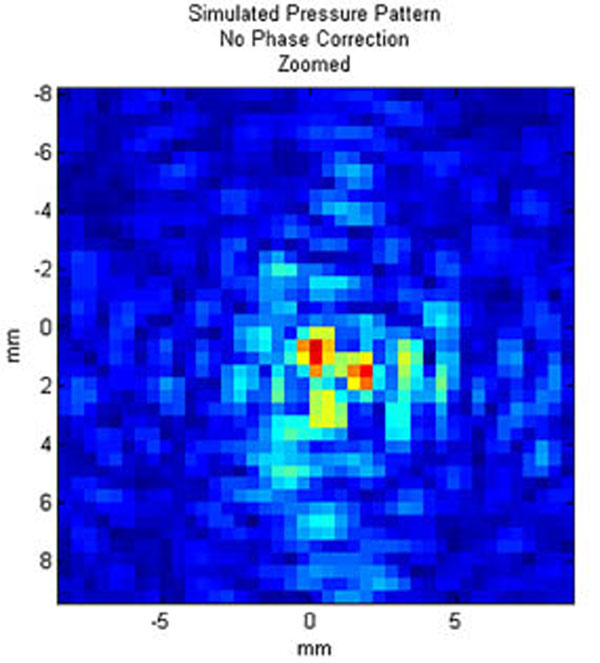
Zoomed-in view of focal spot for pressure pattern shown in 1a.

**Figure 3:**
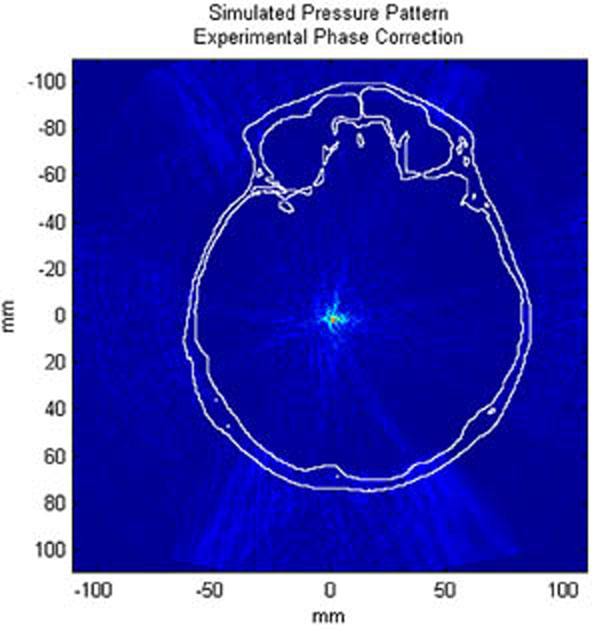
Transverse slice of simulated pressure pattern through the skull with experimentally employed phase correction. Skull is outlined in white

**Figure 4:**
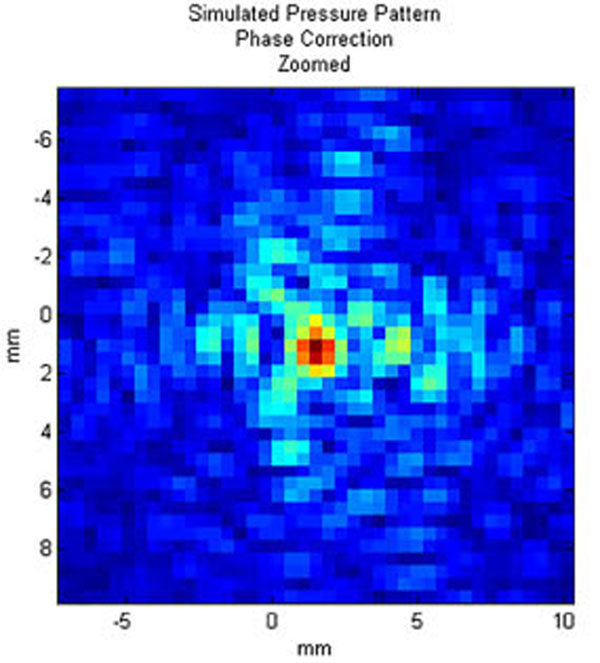
Zoomed-in view of focal spot for pressure pattern shown in 2a.

The average calculation time for each of the sections, including rotation of the model, propagation, and backwards rotation and interpolation of the pressure field, was under 14.5 minutes. Figure 1 shows a transverse slice at the location of peak pressure for one clinical case; Figure 2 shows a zoomed-in view of the focal point. Figures 3-4 present the same pressure field using the experimentally obtained phase corrections.

There is an increase of approximately 25% in peak intensity when phase correction is employed.
